# Anemia as a Potent and Underrecognized Driver of Venous Thromboembolism: A Systematic Review

**DOI:** 10.3390/jcm15020411

**Published:** 2026-01-06

**Authors:** Ghaith K. Mansour, Walaa A. Alshahrani, Lama Alfehaid, Abdulmajeed M. Alshehri, Majed S. Al Yami

**Affiliations:** 1 Department of Pharmaceutical Sciences, College of Pharmacy, Alfaisal University, Riyadh 11533, Saudi Arabia; gkmansour@alfaisal.edu; 2 King Abdullah International Medical Research Center (KAIMRC), Riyadh 11481, Saudi Arabia; alshahraniiwa@gmail.com (W.A.A.); fehaidl@ksau-hs.edu.sa (L.A.); shehriabdul@ksau-hs.edu.sa (A.M.A.); 3 Department of Pharmacy Practice, College of Pharmacy, King Saud bin Abdulaziz University for Health Sciences, Riyadh 11481, Saudi Arabia; 4 King Abdulaziz Medical City, National Guard Health Affairs, Riyadh 14611, Saudi Arabia

**Keywords:** anemia, iron deficiency, vitamin B12, folate deficiency, venous thromboembolism, systematic review

## Abstract

**Background**: Nutritional deficiency anemias—including iron, vitamin B12, and folate deficiencies—are common worldwide and are increasingly recognized as potential contributors to venous thromboembolism (VTE). Mechanistic and epidemiologic data suggest that anemia may promote thrombosis through hypoxia, endothelial activation, reactive thrombocytosis, and hyperhomocysteinemia. However, a focused synthesis of clinical and genetic evidence specifically linking nutritional deficiency anemia to VTE has been lacking. **Methods:** We conducted a systematic search of PubMed and the Cochrane Library from inception to 30 September 2025 to identify studies assessing nutritional deficiency anemia in relation to VTE outcomes. Eligible studies included observational designs, case reports, case series, and Mendelian randomization (MR) analyses. Quality assessment followed the Newcastle–Ottawa Scale (NOS), Joanna Briggs Institute (JBI) checklists, and ROB-MR. The review was registered in PROSPERO (CRD420251235479). **Results:** Seven studies met the inclusion criteria. Observational analytical studies consistently showed that anemia was associated with adverse VTE-related outcomes. Lower hemoglobin predicted higher short-term mortality in acute pulmonary embolism (HR 1.16 per 1 g/dL decrease), increased symptomatic VTE among hospitalized patients (RR 1.94), and greater long-term bleeding and mortality risk in VTE cohorts (HRs 1.41–2.89). Iron-deficiency anemia increased the odds of VTE in population-based data (OR 1.43), and case reports described unprovoked DVT in young adults with moderate to severe anemia. The MR study indicated a potential causal association between anemia traits and thrombosis at unusual anatomical sites (OR 1.446). No study demonstrated a significant association with recurrent VTE. Most analytical studies were rated as good–high quality. **Conclusion:** Across multiple study designs, anemia—particularly iron-deficiency anemia and low baseline hemoglobin—appears to be an underrecognized factor associated with elevated VTE risk and adverse VTE-related outcomes. However, direct evidence for vitamin B12- and folate-deficiency anemia remains limited, and further well-designed prospective studies are required to confirm causality and clarify the contribution of specific nutritional deficiency subtypes, as well as to support integration of anemia assessment into VTE risk models.

## 1. Introduction

Anemia remains one of the most significant global health burdens, affecting an estimated 1.92 billion individuals worldwide and contributing substantially to morbidity, impaired functional capacity, and adverse clinical outcomes [1]. Nutritional deficiency anemias—including iron-deficiency anemia (IDA), vitamin B12-deficiency anemia, and folate-deficiency anemia—constitute the predominant etiologies, with IDA alone accounting for more than two-thirds of all anemia cases and disproportionately affecting women and younger populations [1,2]. These conditions result in reduced hemoglobin concentration and diminished oxygen-carrying capacity, triggering compensatory physiological responses such as increased cardiac output, systemic hypoxia, heightened oxidative stress, and reactive thrombocytosis [3]. Such alterations establish a biologically plausible foundation for a prothrombotic state.

Venous thromboembolism (VTE), comprising deep vein thrombosis (DVT) and pulmonary embolism (PE), is the third most common cardiovascular disorder globally, with an incidence ranging from 100 to 200 per 100,000 individuals annually and substantial rates of recurrence, disability, and mortality [4]. The classical risk factors for VTE—immobilization, malignancy, hormonal therapy, surgery, and inherited thrombophilia—do not fully explain the burden observed in clinical practice. Emerging evidence suggests that nutritional deficiency anemia may serve as an overlooked modifier of VTE risk, supported by epidemiologic, mechanistic, and preliminary genetic data [5,6,7,8,9,10,11,12].

IDA has been linked to hypercoagulability through several distinct mechanisms, including reactive thrombocytosis, platelet hyper-reactivity, increased blood viscosity due to microcytosis, endothelial dysfunction caused by chronic tissue hypoxia, and reduced antioxidant defenses leading to increased oxidative stress and platelet activation [5,6]. Additionally, iron deficiency has been associated with elevated factor VIII levels, which represent a robust and well-established risk factor for thrombosis [7]. Vitamin B12 and folate deficiencies further amplify thrombotic potential by inducing hyperhomocysteinemia, which promotes endothelial injury, increases expression of tissue factor, and disrupts the protein C-mediated anticoagulant pathway [8,9]. Collectively, these mechanisms suggest that nutritional deficiency anemia may contribute to thrombogenesis through multiple converging pathways.

Clinical and epidemiologic studies have increasingly supported this association. Large population-based analyses have demonstrated that individuals with anemia exhibit higher rates of VTE, including DVT and PE, compared with non-anemic populations, even after adjustment for comorbidities and traditional risk factors [10,11]. In particular, IDA has been linked to increased odds of both provoked and unprovoked VTE, and several clinical series have reported VTE events in otherwise healthy young adults presenting with moderate to severe IDA [12]. The presence of anemia in patients with established VTE has also been associated with increased mortality, higher bleeding risk, and worse long-term outcomes [13]. Moreover, recent Mendelian randomization studies provide preliminary genetic-level support for a potential causal relationship between anemia-related traits and thrombotic risk [14].

Despite these important observations, the evidence remains fragmented across heterogeneous study designs, patient populations, and anemia subtypes. No prior systematic review has comprehensively synthesized the association between nutritional deficiency anemia—including iron, vitamin B12, and folate deficiency—and the risk of VTE across observational, descriptive, and genetic studies. Clarifying this relationship is essential for improving risk stratification, refining VTE prediction models, and informing screening and prevention strategies in anemic patients. The present systematic review aims to address this critical gap by evaluating the association between nutritional deficiency anemia and VTE across the totality of available evidence, integrating findings from cohort, case–control, case-based, and genetic studies.

## 2. Materials and Methods

### 2.1. Search Strategy, Study Selection, and Data Extraction

A systematic search was conducted in PubMed and the Cochrane Library to identify studies evaluating the association between iron-deficiency anemia, vitamin B12 deficiency anemia, and folate-deficiency anemia with venous thromboembolism (VTE). The PubMed search strategy combined the terms: (“Anemia” [Mesh] OR “Iron-Deficiency Anemia” [Mesh] OR “Anemia, Vitamin B 12 Deficiency” [Mesh] OR “Folic Acid Deficiency” [Mesh]) AND (“Venous Thromboembolism” [Mesh] OR “Pulmonary Embolism” [Mesh] OR “Deep Vein Thrombosis” [Mesh] OR “Venous Thrombosis” [Mesh]). A similar conceptual strategy was applied to the Cochrane Library using equivalent free-text keywords. The electronic search included all English-language records published from inception to 30 September 2025, consistent with the search settings used by the investigator. Eligible studies included adult participants (≥18 years) and reported an association between nutritional deficiency anemia and VTE outcomes (PE, DVT, or composite VTE). Observational designs were included—prospective or retrospective cohort studies, registry-based analyses, and descriptive studies such as case–control and case series. We excluded secondary evidence (systematic reviews, meta-analyses, or guidelines), non-eligible designs (animal studies, conference abstracts), studies outside the scope (pediatric populations, non-nutritional anemia types such as hemolytic disorders, or lacking VTE outcomes), and studies in non-English languages.

### 2.2. Quality Assessment and Risk of Bias

The methodological quality and risk of bias of the included studies were assessed according to the design-specific, internationally accepted appraisal tools. Observational analytical studies (cohort and case–control) were evaluated using the Newcastle–Ottawa Scale (NOS), which assesses three domains: Selection (0–4 stars), Comparability (0–2 stars), and Outcome (0–3 stars), with a maximum score of 9. Based on established criteria, studies scoring 7–9 stars were classified as high quality, 5–6 stars as moderate quality, and ≤4 stars as low quality. Four studies in this review [15,16,17,18] were eligible for NOS assessment, all demonstrating good to high methodological quality [19].

Studies that were not eligible for NOS due to their descriptive or non-comparative design were appraised using the appropriate validated tools. The case report [18] and case series [20] were assessed using the Joanna Briggs Institute (JBI) Critical Appraisal Checklists for Case Reports and Case Series, respectively. These tools evaluate clarity of clinical presentation, diagnostic accuracy, completeness of patient information, intervention detail, and outcome reporting. Based on JBI criteria, the case report demonstrated moderate quality, while the case series demonstrated low to moderate quality due to limited sample size and lack of consecutive recruitment [21].

The Mendelian randomization study [22] was evaluated using the Risk of Bias in Mendelian Randomization Studies (ROB-MR) tool, which examines instrument strength, independence from confounders, absence of horizontal pleiotropy, heterogeneity, and robustness of sensitivity analyses. This study demonstrated a low risk of bias, supported by strong genetic instruments and multiple sensitivity analyses (MR-Egger, weighted median, MR-PRESSO) [23].

All assessments were conducted independently by two reviewers, and any discrepancies were resolved through discussion and consensus. No study was excluded on the basis of methodological quality alone, and the interpretation of results accounted for the inherent strengths and limitations of each study design. A comprehensive summary of all risk-of-bias evaluations and quality ratings is presented in Table 1. The study was conducted according to PRISMA guidelines and registered in the International Prospective Register of Systematic Reviews (PROSPERO) with the registration number CRD420251235479. The PRISMA Checklist is in the Supplementary Material (Table S1).

### 2.3. Data Synthesis and Analysis

A meta-analysis was not feasible due to substantial heterogeneity across the included studies. The designs (cohort, case–control, randomized trial substudy, Mendelian randomization, case series, and case report), populations, definitions of nutritional deficiency anemia, VTE outcome measures, and follow-up durations varied widely. Because of these differences, the effect estimates (HRs, RRs, ORs, and adjusted measures) were extracted and presented exactly as reported in the original publications, without statistical transformation or standardization.

A structured narrative synthesis was therefore conducted following PRISMA 2020 guidelines. Analytical studies were summarized based on effect direction and magnitude, whereas case-based studies were synthesized descriptively to identify recurrent clinical patterns without inferring causality. The Mendelian randomization study was evaluated separately, given its distinct genetic instrumental-variable approach. Importantly, none of the included studies shared overlapping patient cohorts or utilized the same datasets, ensuring independence of results. No subgroup or sensitivity analyses were performed due to the limited number and heterogeneity of studies.

## 3. Results

### 3.1. Search Results and Study Characteristics

**Figure 1:**
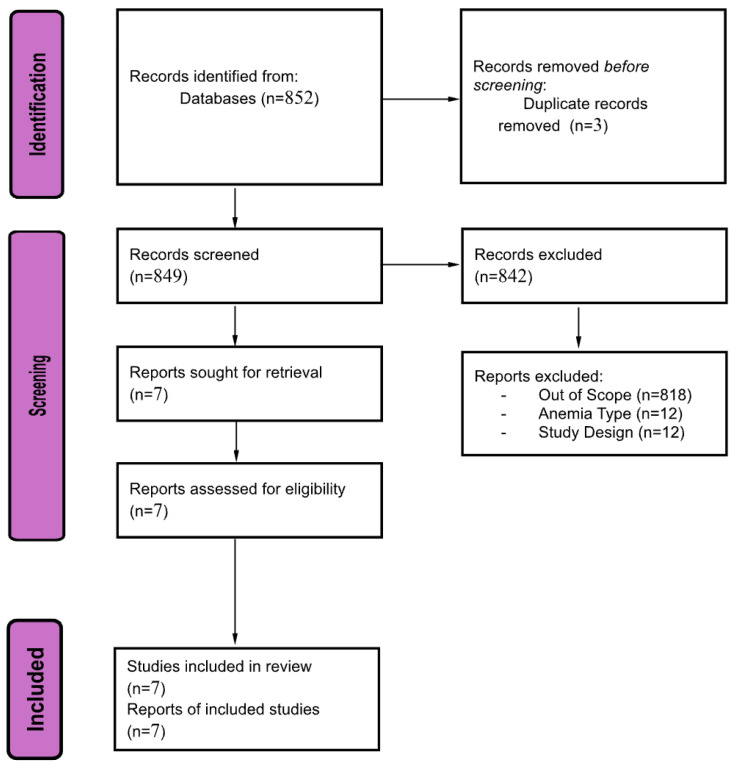
PRISMA flow diagram for selection of studies for the systematic review.

A total of 852 records were identified through the database search. After the removal of 3 duplicate records, 849 records proceeded to title and abstract screening. Of these, 842 records were excluded for not meeting the eligibility criteria. The remaining 7 reports were retrieved for full-text assessment, all of which met the inclusion criteria. Ultimately, seven studies were included in the systematic review, as shown in (Figure 1). The systematic search identified seven eligible studies examining the association between anemia and venous thromboembolism (VTE). These included four observational analytical studies (three cohort studies and one case–control study), one Mendelian randomization study, one case series, and one case report. The observational studies varied in sample size, ranging from 764 to over 3000 patients, while the genetic study included multiple large-scale GWAS datasets. Two descriptive clinical reports described individual or paired cases of IDA-associated DVT or PE. Across studies, anemia definitions varied (hemoglobin thresholds or ICD coding), and outcomes included mortality, symptomatic VTE, recurrent VTE, major bleeding, or site-specific thrombotic events. It is important to note that the authors defined anemia based on hemoglobin level only without investigating its underlying etiology. This could explain that nutritional deficiency anemia has no direct association with causing VTE.

Follow-up periods ranged from 77 days to over three years, as shown in Table 2. The methodological quality of the included studies was generally acceptable across designs, as summarized in Table 2.

### 3.2. Summary of the Included Studies

Jiménez 2009 et al. conducted a prospective cohort study of 764 patients with acute pulmonary embolism and found that lower hemoglobin levels were significantly associated with worse short-term outcomes. Patients in the lowest hemoglobin quartile had a markedly reduced 3-month survival rate of 75% compared with 86–91% in higher quartiles. Each 1 g/dL decrease in hemoglobin increased the risk of all-cause mortality by 16% (HR 1.16, 95% CI 1.05–1.28), and anemia was also linked to a higher unadjusted risk of fatal PE [15].

Hung 2015 et al. conducted a nationwide population-based case–control study including 2522 VTE cases and 12,610 controls. Prior iron-deficiency anemia was independently associated with a 43% higher odds of developing VTE (adjusted OR 1.43, 95% CI 1.10–1.87). IDA was also specifically associated with deep-vein thrombosis (OR 1.43, 95% CI 1.08–1.90), although no significant relationship was observed with isolated pulmonary embolism [16].

Chi 2018 et al. conducted a secondary analysis of the APEX trial including 6861 acutely ill hospitalized patients and found that low baseline hemoglobin significantly increased the risk of symptomatic VTE despite prophylaxis. Low hemoglobin was associated with higher rates of total symptomatic VTE (RR 1.94, 95% CI 1.27–2.98), DVT (RR 2.29, 95% CI 1.12–4.68), and non-fatal PE (RR 2.63, 95% CI 1.22–5.65). After adjustment, anemia remained an independent predictor of VTE (adjusted OR 1.71, 95% CI 1.09–2.69) [11].

Yamashita 2019 et al. conducted a multicenter prospective cohort study of 3012 VTE patients and demonstrated that baseline anemia was strongly associated with long-term bleeding and mortality outcomes. Moderate/severe anemia significantly increased the risk of major bleeding (HR 1.91, 95% CI 1.42–2.58) and all-cause mortality (HR 2.89, 95% CI 2.45–3.42), while mild anemia also increased bleeding risk (HR 1.41, 95% CI 1.00–1.98). Anemia was not associated with recurrent VTE (HR 1.05, 95% CI 0.76–1.45) [17].

Ezeh 2021 et al. described a 72-year-old woman with severe iron-deficiency anemia who developed recurrent bilateral PE and DVT. Management with blood transfusion, iron supplementation, and anticoagulation stabilized her hemoglobin, after which no further thromboembolic events occurred, highlighting severe IDA as a possible driver of recurrent VTE [18].

Yadav 2023 et al. reported two young adults with moderate iron-deficiency anemia who presented with unprovoked lower-limb DVT. Both exhibited reactive thrombocytosis and lacked alternative provoking factors. Correction of anemia along with anticoagulation led to clinical improvement, suggesting that IDA may contribute to unprovoked DVT even in low-risk individuals [24].

Yu 2025 et al. applied a two-sample Mendelian randomization approach using large GWAS datasets and found a potential causal relationship between anemia and thrombosis. Genetic predisposition to anemia increased the risk of thrombosis in unusual anatomical sites (OR 1.446, 95% CI 1.104–1.895), while aplastic anemia showed a mild association with overall VTE (OR 1.065, 95% CI 1.003–1.131). No strong causal effect was detected for classic DVT or PE [20].

### 3.3. Outcomes

Across all included studies, anemia was consistently associated with adverse thrombotic or clinical outcomes, although the magnitude and nature of risk varied by study design. Nevertheless, study design, patient groups, and anemia classifications demonstrated significant variation across these cohorts, reflecting the importance of interpreting these findings with caution. Observational studies demonstrated that anemia predicted higher short-term mortality in acute PE (HR 1.16 per 1 g/dL decrease), greater risk of symptomatic VTE in hospitalized medically ill patients (RR 1.94), and increased risk of long-term major bleeding and mortality in established VTE cohorts (HRs 1.41–2.89). IDA specifically increased the odds of VTE in population-level data (OR 1.43) and was linked to unprovoked DVT in young individuals in descriptive reports. Genetic evidence supported a potential causal relationship between anemia and thrombosis, particularly in unusual sites (OR 1.446). All clinical outcomes from the included studies are shown in Table 3.

## 4. Discussion

This systematic review provides comprehensive evidence linking nutritional deficiency anemia—including iron-deficiency anemia, vitamin B12 deficiency, and folate-deficiency anemia—to an increased risk of venous thromboembolism (VTE). However, only one study clearly demonstrates an independent effect of iron deficiency on the risk of VTE [16]. In several other key studies, the specific type or etiology of anemia was not further defined [11,15,17,24]. Furthermore, one study analyzed genetic risk profiles for a broad spectrum of anemias, including non-nutritional forms such as hereditary hemolytic anemia and aplastic anemia [20]. Vitamin B12 and folate-deficiency anemias may contribute to thrombogenesis through hyperhomocysteinemia-mediated endothelial injury and disruption of anticoagulant pathways [8,9]. Iron deficiency promotes reactive thrombocytosis and platelet hyper-reactivity, increases platelet activation markers, and elevates factor VIII levels—an established VTE risk factor [7]. Microcytosis in IDA may impair blood flow and contribute to venous stasis, while chronic hypoxia amplifies endothelial dysfunction and oxidative stress, resulting in a hypercoagulable state [6]. Consistently, deficiencies in vitamin B12 and folate elevate homocysteine levels, leading to endothelial activation, tissue-factor expression, and reduced protein C activity [8,9]. These mechanisms strongly reinforce the epidemiologic associations observed in clinical studies.

Our findings align with broader epidemiologic evidence. The Swedish–Danish blood donor cohort demonstrated that individuals with subnormal hemoglobin experienced up to a two-fold increase in VTE risk after adjustment for confounders [10]. Similarly, the GARFIELD-VTE registry showed that anemic VTE patients had significantly higher long-term mortality and major bleeding risk compared with non-anemic patients, underscoring the prognostic significance of anemia in thrombosis [13]. Additionally, preliminary genetic evidence from Mendelian randomization suggests a potential causal effect of anemia-related traits on thrombotic susceptibility [20]. Clinically, these findings highlight anemia as an underrecognized VTE risk modifier. Routine assessment of hemoglobin, ferritin, vitamin B12, and folate levels may help identify individuals at increased thrombotic risk. Correction of nutritional anemia could represent a modifiable therapeutic target, although prospective trials are required to confirm benefit. Importantly, widely used VTE risk-assessment models currently omit anemia-related variables, despite increasing evidence supporting their relevance [11,25].

This review has notable strengths. It is the first to synthesize evidence specifically on nutritional deficiency anemia and VTE across observational, descriptive, and genetic methodologies. Use of validated appraisal tools (NOS, JBI, ROB-MR) enhances confidence in study quality. Integrating mechanistic and genetic research provides a multidimensional understanding of the anemia–VTE connection. However, several limitations must be acknowledged. Only a limited number of high-quality analytic studies exist, with significant heterogeneity in anemia definitions and population characteristics. Observational designs are subject to confounding, and case-based evidence provides low-level support. Only one Mendelian randomization study was identified, limiting the strength of causal inference. The current evidence on nutritional deficiency anemia remains limited, particularly regarding vitamin B12- and folate-deficiency anemia. Moreover, most included studies did not account for the underlying cause of anemia or other critical risk factors for VTE, such as renal failure and cancer. Therefore, the observed association should be interpreted with caution, as anemia in many included populations may represent a surrogate marker of disease severity rather than an independent causal factor. Future research should focus on large, well-designed prospective cohorts that clearly differentiate anemia subtypes and quantify their independent contributions to VTE risk.

## 5. Conclusions

In conclusion, the available evidence suggests that nutritional deficiency anemia may contribute to a clinically relevant yet underappreciated determinant of venous thromboembolic risk across heterogeneous study designs. Despite the biologically plausible pathways linking anemia to prothrombotic states, the current literature remains insufficient to establish definitive causality. Accordingly, rigorously designed prospective investigations and targeted interventional studies are warranted to delineate this relationship more clearly and to determine whether integrating anemia-related parameters into existing VTE risk-assessment frameworks could enhance prognostic accuracy and clinical decision-making.

## Figures and Tables

**Table 1:** Quality assessment of included Studies.

Observational Studies Using the Newcastle–Ottawa Scale (NOS)					
Study (Author, Year)	Selection (0–4)	Comparability (0–2)	Outcome (0–3)	Total (0–9)	Quality
Jiménez et al., 2009 [15]	4	2	2	8/9	Good
Hung SH et al., 2015 [16]	4	2	2	8/9	Good
Chi et al., 2018 [11]	4	2	2	8/9	Good
Yamashita et al., 2019 [17]	4	2	3	9/9	High
Non-NOS-Eligible Studies Using JBI and ROB-MR Tools					
Study (Author, Year)	Tool Used	Key Assessment Criteria	Summary of Assessment	Overall Quality	
Ezeh, 2021 * [18]	JBI Case Report Checklist	Clinical history; Presentation; Diagnostic tests; Intervention; Follow-up; Outcomes reporting	Clear clinical details; Diagnostic steps documented; Outcomes described; Limited by single-patient design	Moderate	
Yadav, 2023 ** [24]	JBI Case Series Checklist	Inclusion clarity; Patient characteristics; Diagnostic reliability; Consecutive recruitment; Outcome reporting	Patient data clearly described; Very small sample (n = 2); No consecutive recruitment reported	Low–Moderate	
Yu, 2025 *** [20]	ROB-MR Tool	Instrument strength; Independence; Exclusion restriction; Pleiotropy; Sensitivity analyses	Strong instruments; Extensive sensitivity analyses (MR-Egger, WM, MR-PRESSO); Low pleiotropy risk	Low Risk of Bias	

* Case Report, ** Case Series, *** Mendelian Randomization.

**Table 2:** Baseline Characteristics of Included Studies.

Study (Author, Year)	Study Design	Setting (Country/Registry)	Type of Anemia	Population	Mean/Median Age (Years)	Follow-Up Duration
Jiménez et al., 2009 [15]	Prospective cohort	Spain	General anemia (defined by Hb only; not by type)	764 acute symptomatic PE patients	The age ranged from 20 to 96 years *	3 months
Hung SH et al., 2015 [16]	Population-based case–control	Taiwan	Iron Deficiency Anemia (IDA)	2522 VTE cases + 12,610 controls	For the overall study sample, the mean age was 62.5 ± 16.9 years, and mean ages were 62.5 ± 17.0 years for cases and 62.9 ± 16.8 years for controls.	NA
Chi et al., 2018 [11]	Randomized trial substudy (APEX Trial)	Multicenter international RCT (USA, Canada, United Kingdom)	Low hemoglobin (type not specified)	6861 patients with baseline Hb and follow-up	mean (SD) 77.0 (8.5)	77 days
Yamashita et al., 2019 [17]	Multicenter observational cohort	Japan	Anemia (mild–moderate–severe) (type not specified)	3012 acute symptomatic VTE patients	Moderate/Severe Anemia → 68.2 ± 15.9 Mild Anemia → 69.9 ± 14.4 No Anemia → 65.2 ± 15.1	Median 1219 days (Interquartile range (IQR) 847–1765)
Ezeh et al., 2021 [18]	Case report	United States	Iron Deficiency Anemia (IDA)	One patient with severe IDA and recurrent VTE	72 years old	NA
Yadav et al., 2023 [24]	Case series	India	Moderate anemia + reactive thrombocytosis (type not specified)	Two young patients with unprovoked DVT and moderate anemia	35 years old and 45 years old.	NA
Jieni Yu et al., 2025 [20]	Mendelian Randomization	China, United Kingdom, Finland	Anemia. IDA, Aplastic Anemia (AA) Hereditary Hemolytic Anemias (HHA)	GWAS datasets: anemia (5259), IDA (12,317), AA (4128), VTE (21,021)	NR	NA

*: The article did not report Mean/Median Age (Years), only a range. NR: The manuscript does NOT report age at all, neither mean nor median, and it has no age subgroup classification. NA: Not available.

**Table 3:** Clinical Outcomes.

Study (Author, Year)	Outcome	Effect Estimate (95% CI)	*p*-Value
Jiménez (2009) [15]	All-cause Mortality	- All patients: adjusted HR 0.86, 95% CI 0.78 to 0.95 - After excluding cancer patients: adjusted HR 0.81; 95% CI, 0.66 to 0.99	- All patients: *p* = 0.006 - After excluding cancer patients: *p* = 0.04
	Fatal PE	Unadjusted HR 1.19 (1.04–1.37)	NR
Hung (2015) [16]	VTE (All-cause)	Adjusted OR 1.43 (1.10–1.87)	<0.05
	DVT (subgroup)	OR 1.43 (1.08–1.90)	<0.05
	PE (subgroup)	OR 1.10 (0.63–1.92)	NR
Chi (2018) [11]	Symptomatic VTE	RR 1.94 (1.27–2.98)	0.002
	Symptomatic DVT	RR 2.29 (1.12–4.68)	0.019
	Non-fatal PE	RR 2.63 (1.22–5.65)	0.010
	Adjusted VTE Risk	Adjusted OR 1.71 (1.09–2.69)	0.020
Yamashita (2019) [17]	Recurrent VTE	Mild anemia: Adjusted HR 0.92 (95% CI 0.63–1.32) Moderate/severe anemia: HR 1.05 (95% CI 0.76–1.45)	Mild anemia: 0.65 Moderate/severe anemia: 0.77
	Long-term Mortality	Mild anemia: Adjusted HR1.21 (0.98–1.49) Moderate/severe: Adjusted HR 1.68 (1.40–2.01)	Mild anemia: 0.08 Moderate/severe: <0.001
	Major Bleeding	Mild anemia: Adjusted HR 1.41 (1.00–1.98) Moderate/severe: Adjusted HR 1.91 (1.42–2.58)	Mild anemia: 0.048 Moderate/severe: <0.001
Ezeh (2021) [18]	PE + DVT Clinical Course	No effect estimate reported	NR
Yadav (2023) [24]	Unprovoked DVT	No effect estimate reported	NR
Yu (2025) [20]	Genetic VTE Risk (overall VTE)	OR 1.065 (1.011–1.122)	0.040
	Genetic Risk (unusual-site embolism)	OR 1.446 (1.104–1.895)	0.007
	VTE	OR 1.2608 (0.9860–1.6122)	0.0646
	PE	OR 1.2453 (0.9909–1.5651)	0.0599
	DVT	OR 1.3507(0.9822–1.8584)	0.0644

NR: Not Reported.

## Data Availability

All data generated or analyzed during this study are included in this article.

## Supplementary Materials

The following supporting information can be downloaded at https://www.mdpi.com/article/10.3390/jcm15020411/s1. Table S1: PRISMA Checklist [26].

## Abbreviations

The following abbreviations are used in this manuscript:

IDA	Iron-deficiency anemia
VTE	Venous thromboembolism
DVT	Deep vein thrombosis
PE	Pulmonary embolism
Hb	Hemoglobin
RCT	Randomized controlled trial
MR	Mendelian randomization
GWAS	Genome-wide association study
NOS	Newcastle–Ottawa Scale
JBI	Joanna Briggs Institute
ROB-MR	Risk of Bias in Mendelian Randomization
OR	Odds ratio
RR	Risk ratio
HR	Hazard ratio
aOR	Adjusted odds ratio
PRISMA	Preferred Reporting Items for Systematic Reviews and Meta-Analyses
PROSPERO	International Prospective Register of Systematic Reviews
AA	Aplastic anemia
HHA	Hereditary hemolytic anemias
SD	Standard deviation
IQR	Interquartile range
NR	Not reported
NA	Not available
